# Phenotypic screening of the ‘Kurz-box’ of chemicals identifies two compounds (BLK127 and HBK4) with anthelmintic activity *in vitro* against parasitic larval stages of *Haemonchus contortus*

**DOI:** 10.1186/s13071-019-3426-7

**Published:** 2019-04-30

**Authors:** Linh Thuy Nguyen, Thomas Kurz, Sarah Preston, Hjoerdis Brueckmann, Beate Lungerich, H. M. P. Dilrukshi Herath, Anson V. Koehler, Tao Wang, Lenka Skálová, Abdul Jabbar, Robin B. Gasser

**Affiliations:** 1 Department of Veterinary Biosciences, Melbourne Veterinary School, Faculty of Veterinary and Agricultural Sciences, The University of Melbourne, Parkville, VIC Australia; 2 Department of Biochemical Sciences, Charles University, Faculty of Pharmacy, Hradec Králové, Czech Republic; 3 Institute of Pharmaceutical and Medicinal Chemistry, Heinrich-Heine University Düsseldorf, Düsseldorf, Germany; 4 Faculty of Science and Technology, Federation University, Ballarat, VIC Australia

**Keywords:** *Haemonchus contortus*, Phenotypic screening, Anthelmintic, Larval motility and development *in vitro*

## Abstract

**Background:**

Due to anthelmintic resistance problems, there is a need to discover and develop new drugs for the treatment and control of economically important and pathogenic nematodes of livestock animals. With this focus in mind, we screened 236 compounds from a library (called the ‘Kurz-box’) representing chemically diverse classes such as heterocyclic compounds (e.g. thiazoles, pyrroles, quinolines, pyrimidines, benzo[1,4]diazepines), hydoxamic acid-based metalloenzyme inhibitors, peptidomimetics (bis- and tris-pyrimidoneamides, alkoxyamides) and various intermediates on *Haemonchus contortus*, one of the most important parasitic nematodes of ruminants.

**Methods:**

In the present study, we tested these compounds, and measured the inhibition of larval motility and development of exsheathed third-stage (xL3) and fourth-stage (L4) larvae of *H. contortus* using an optimised, whole-organism phenotypic screening assay.

**Results:**

Of the 236 compounds, we identified two active compounds (called BLK127 and HBK4) that induced marked phenotypic changes in the worm *in vitro*. Compound BLK127 induced an ‘eviscerated’ phenotype in the xL3 stage and also inhibited L4 development. Compound HBK4 exerted a ‘curved’ phenotype in both xL3s and L4s.

**Conclusions:**

The findings from this study provide a basis for future work on the chemical optimisation of these compounds, on assessing the activity of optimised compounds on adult stages of *H. contortus* both *in vitro* and *in vivo* (in the host animal) and against other parasitic worms of veterinary and medical importance.

**Electronic supplementary material:**

The online version of this article (10.1186/s13071-019-3426-7) contains supplementary material, which is available to authorized users.

## Background

Parasitic worms (helminths) cause a major disease burden on humans and animals worldwide. A highly pathogenic parasitic nematode of livestock animals is *Haemonchus contortus*, also known as the barber’s pole worm. This species is recognised as one of the most economically important parasites of ruminants, because it impairs weight gain and productivity, and causes disease and mortality, particularly in young animals [1]. Sheep and goats with large burdens of *H. contortus* develop anaemia and can die in the absence of effective treatment.

Although available anthelmintics including benzimidazoles, imidazothiazoles, macrocyclic lactones, salicylanilides, amino-acetonitrile derivatives or spiroindoles [1] are used for the treatment of parasitic nematodes, chemical control is becoming less effective due to the occurrence of resistance to one or multiple drugs. The high genetic diversity of *H. contortus* gives rise to the rapid selection of resistant worms, whose survival favours the spread of alleles bearing drug resistance traits to progeny [2–4]. Moreover, the regular, if not excessive use of chemical treatment and management practices contribute to increased selection pressure in subsequent worm generations. Drug resistance is now very widespread in parasitic nematodes of particularly small ruminants [1, 5], and there are reports of resistance to, or reduced efficacy of, some recently commercialised anthelmintics, such as monepantel or derquantel [6, 7]; there is also an increased prevalence of multi-drug-resistant strains [5, 8].

Even though non-chemical methods for parasite control in livestock animals (e.g. nutrition or vaccines) can reduce the reliance on the use of chemicals and are environmentally friendly, none of these methods appear yet sufficiently effective without complementary anthelmintic treatment measures [9]. In order to reduce the burden caused by parasites, such as *H. contortus*, in small ruminants, control programmes can be based on integrated parasite management [1], which takes into account economic factors including epidemiology, resistance status as well as animal production and management systems. Nonetheless, anthelmintic treatment is usually central to parasite control.

Therefore, the discovery of novel chemical entities with unique modes of action against drug-resistant nematodes of livestock is critical. In this context, we have commenced a programme to screen several distinct compound libraries [10–16] against *H. contortus*, a representative strongylid nematode, using a whole-organism phenotypic screening technique established in our laboratory [10]. In the present study, we expand this work by screening a set of compounds (*n* = 236) representing distinct classes of chemicals, including heterocyclic compounds (e.g. thiazoles, pyrroles, quinolines, pyrimidines, benzimidazoles, benzo[1,4]diazepines), hydoxamic acid-based metalloenzyme inhibitors, peptidomimetics (bis- and tris-pyrimidoneamides, alkoxyamides) and various intermediates. The aims of the present study were to (i) undertake a primary screen of the chemicals against exsheathed third-stage (xL3) larvae and identify active (‘hit’) compound/s; (ii) assess the activity and potency of active compounds at inhibiting xL3 and L4 motility and L4 development in a dose–response assay; and (iii) characterise the non-wild-type phenotypes of treated larvae.

## Methods

### Procurement of *H. contortus*

The Haecon-5 strain (Australia, cf. [17]) of *H. contortus* was maintained in experimental sheep as described previously [10], in accordance with institutional animal ethics guidelines (permit no. 1613878; The University of Melbourne, Australia). L3s were produced from *H. contortus* eggs by incubating humidified faeces from infected sheep at 27 °C for 1 week and stored for ≤ 3 months [10]. To produce xL3s, L3s were exposed to 0.15% (v/v) of sodium hypochlorite (NaClO) for 20 min at 37 °C [10], washed five times in sterile physiological saline and cultured in Luria Bertani medium (LB) supplemented with final concentrations of 100 IU/ml of penicillin, 100 µg/ml of streptomycin and 2 µg/ml of amphotericin (LB*). To produce L4s, xL3s were incubated for 7 days at 38 °C and 10% (v/v) CO_2_, when ≥ 80% of xL3s had developed to the L4 stage.

### Preparation of compounds for screening

The compound library (designated ‘Kurz-box’) containing 236 chemicals was assembled and curated by two of the authors (TK and BL) at the Institute of Pharmaceutical and Medicinal Chemistry, Heinrich-Heine-University Düsseldorf, Germany. Individual compounds were dissolved in 100% dimethyl sulfoxide (DMSO) to achieve stock concentrations of 20 mM. Individual compounds were then diluted in LB* and tested for activity against *H. contortus*. The synthesis of the ‘hit’ compounds is given in Additional file 1.

### Screening of compounds for their effect on xL3 motility and L4 development

The whole-organism screening assay, developed by Preston et al. [10], was used to evaluate the effect of compounds on the motility of xL3s of *H. contortus*. In the primary screen, xL3 motility was assessed for each compound (at the final concentration of 20 µM and using three technical replicates) as described previously [10]. Two commercial drugs, monepantel (Zolvix, Novartis Animal Health, Switzerland) and moxidectin (Cydectin, Virbac, France), were used as the positive controls (at the final concentration of 20 µM), and LB* + 0.5% DMSO was used as the negative control. Following an incubation period of 72 h (38 °C, 10% (v/v) CO_2_), a 5 s video recording was taken of each well to capture the motility of xL3s. The plates were then incubated for 4 more days to observe the effect of individual compounds on the development of L4s. After 7 days, a 5 s video was taken for each well. Then, worms were fixed with 50 µl of 1% iodine; L4s were identified microscopically (magnification of 20×) based on the presence of a well-developed pharynx characteristic of *H. contortus* [18] and counted. Length and width of L4s (*n* = 30) were measured and assessed for phenotypic changes using the software program ImageJ (National Institutes of Health, Bethesda, MD, USA). Width was measured at the level of nerve-ring (cf. [19]). Results were expressed as mean ± standard error of the mean (SEM). The number of L4s was expressed as a percentage of the total number of worms counted. The one-way analysis of variance (ANOVA) and Dunnett’s multiple comparison tests were used to compare the effect of compounds on L4 development in comparison to the negative control. If the compound reduced the xL3 motility by > 70% and/or induced phenotypic changes compared with the negative control after 7 days, it was recorded as a ‘hit’ compound.

### Dose–response assessments of identified active compounds on xL3 and L4 motility, and L4 growth and development

The motility of xL3s was assessed in an 18-point dose–response curve (two-fold serial dilutions; from 100 µM to 0.00076 µM). On each 96-well plate, test compounds and the positive controls (monepantel and/or moxidectin) were arrayed in triplicate. Six wells were used for the negative control (LB* + 0.5% DMSO) on each plate. A 5 s video recording was taken of each well after 24 h, 48 h and 72 h [10]. The culture plates were then incubated for 4 more days at 38 °C, 10% (v/v) CO_2_. After a total of 7 days, a 5 s video recording was taken of each well, and motility was recorded using the motility algorithm [10], after which the worms were fixed with 50 µl of 1% iodine. L4 development was assessed as described [10].

The motility of L4s was evaluated using the same protocol as for xL3s [10]. The motility was measured after 24, 48 and 72 h of incubation of L4s with each active compound (triplicate). At the end of L4 motility assay, i.e. after 72 h, worms were observed using light microscope (DP26 camera, Olympus) to determine the structural changes induced by the compounds.

Compound concentrations were transformed using the equation x = log10 (concentration in µM) and a log(inhibitor) *versus* response - variable slope (four parameter) equation in GraphPad Prism v.7.04 was used to calculate the half maximum inhibitory concentration (IC_50_), where possible.

## Results

### Identification of two active compounds with characteristic phenotypic changes in *H. contortus*

**Fig. 1:**
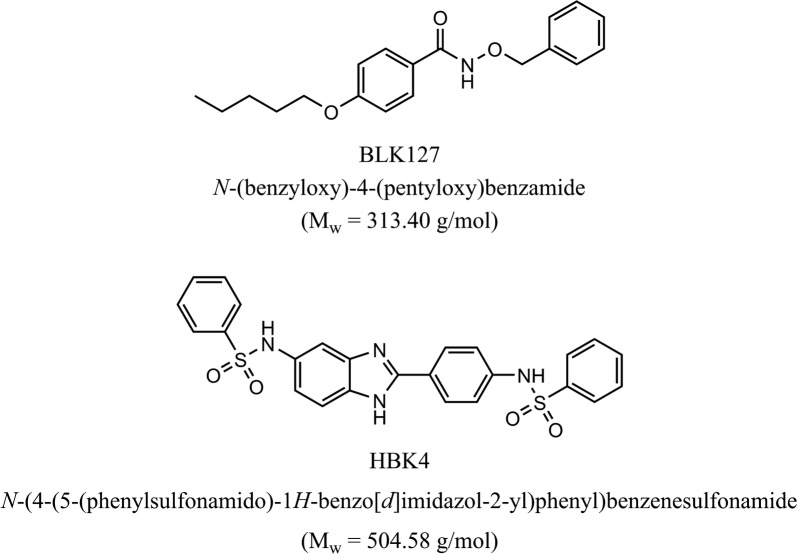
Chemical structure, IUPAC name and molecular weight (M_w_) of the two compounds that were recorded to affect *Haemonchus contortus* in the present study

**Fig. 2:**
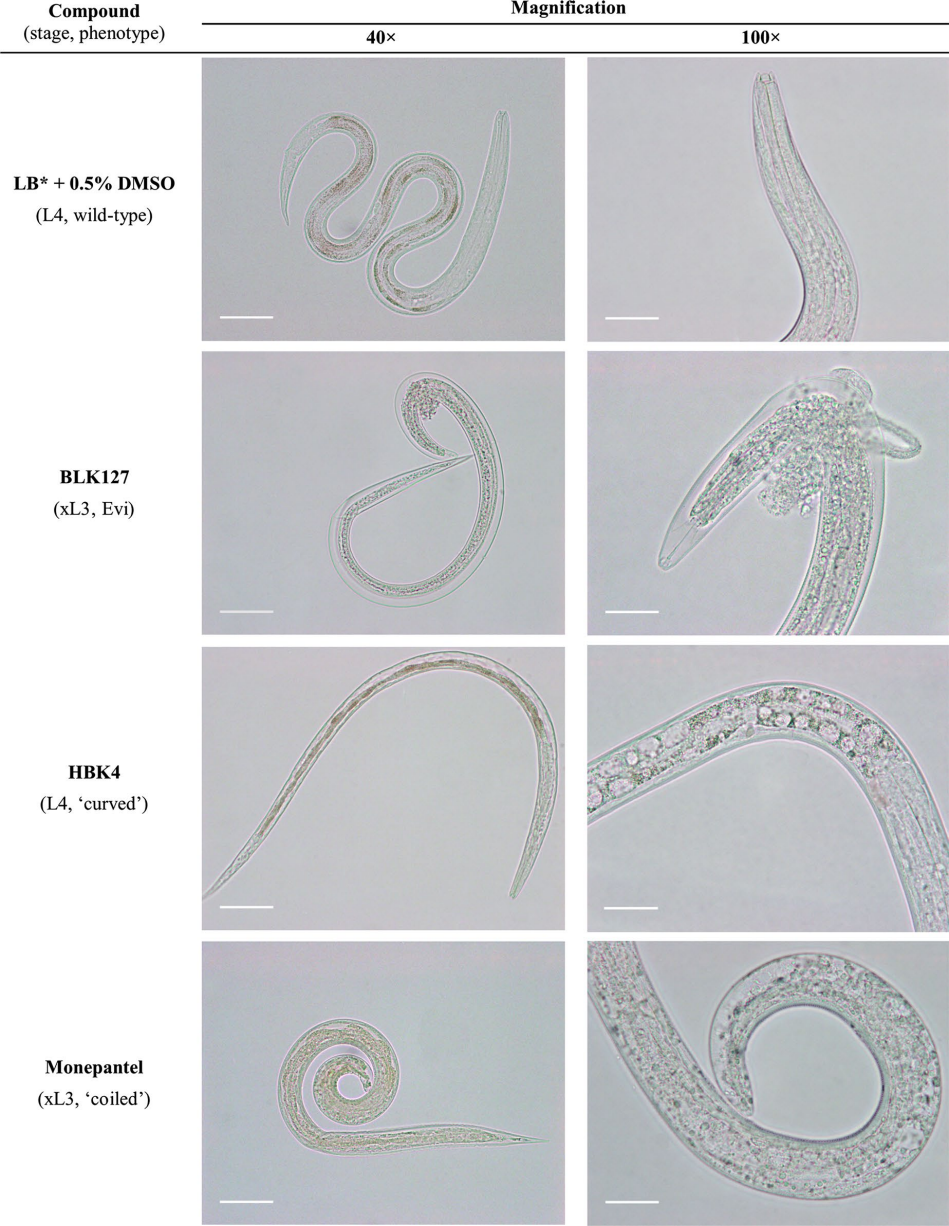
Light microscopy images of different phenotypes of exsheathed third-stage larvae (xL3) or developed fourth-stage larvae (L4) of *Haemonchus contortus* 7 days following exposure of xL3s to 20 µM of compound BLK127, HBK4, monepantel (positive control) or LB* + 0.5% DMSO (negative control). The details of the developed pharynx in the negative control, anterior protrusion in the ‘eviscerated’ (Evi) phenotype and presence of vacuoles in the ‘curved’ phenotype are shown. Scale-bars are 50 µm and 20 µm for 40× and 100× magnification, respectively

In the primary screen of 236 chemicals, none of the compounds inhibited xL3 motility by > 70% after 72 h. However, incubation of culture plates for 4 more days revealed that two compounds induced phenotypic changes in the larvae (Fig. 1, Additional file 1). Compound BLK127 induced an anterior protrusion in xL3s (treated for 7 days); compound HBK4 induced a ‘curved’ phenotype in xL3s (7 days) and L4s (24 h) (Fig. 2).

The phenotypic changes recorded by video in xL3s after 7 days were examined further by light microscopy. A detailed examination of BLK127-treated xL3s revealed an ‘eviscerated’ (Evi) phenotype, consistent with that described by Jiao et al. [20].

Larvae with an Evi phenotype retained their old cuticle, and some of the xL3s with a protrusion had a developed pharynx. However, the severe morphological damage induced by compound BLK127 appeared not to allow larvae to moult to the next stage and resulted in death of the larvae. During the physiological process of ecdysis, the old cuticle breaks approximately at the level of the excretory pore, and the cuticle swells and becomes distorted in this region prior to rupturing [19]. The xL3s exposed to BLK127 gradually (over a period of 72 h) eviscerated and released fluids *via* the excretory pore (108.4 ± 1.2 µm, *n* = 30). The expelled mass extruded through the rupture in the cuticle, and the protrusion was located 80.7 ± 1.5 µm from the anterior tip of the xL3 stage (*n* = 30).

**Fig. 3:**
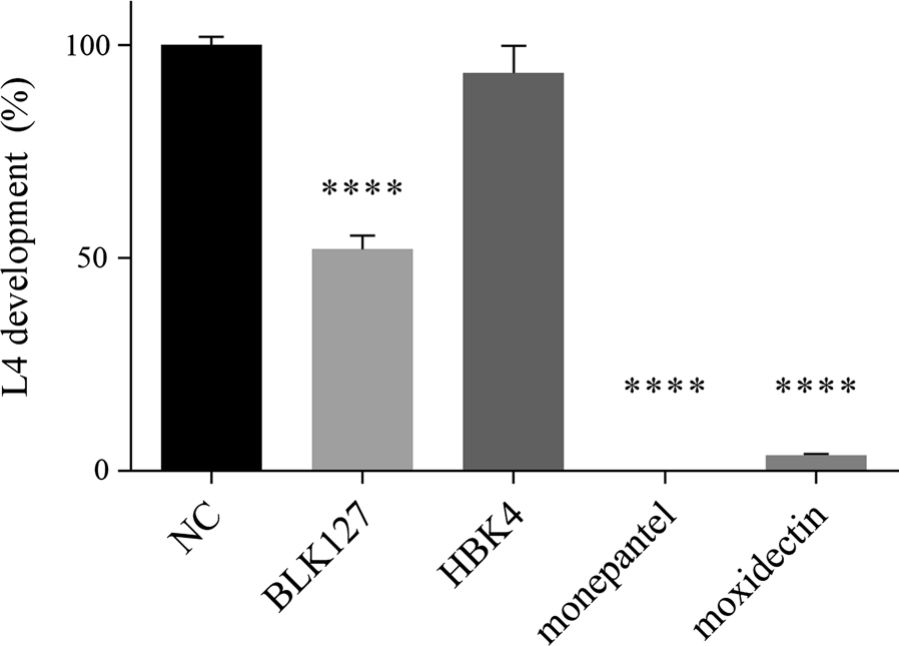
Development of fourth-stage larvae (L4s) (%) after 7 days of exposure to 20 µM of individual compounds; **** denotes significance: *P* < 0.0001 compared to the negative control (NC) LB* + 0.5% DMSO based on one-way ANOVA and Dunnett’s multiple comparison test

**Fig. 4:**
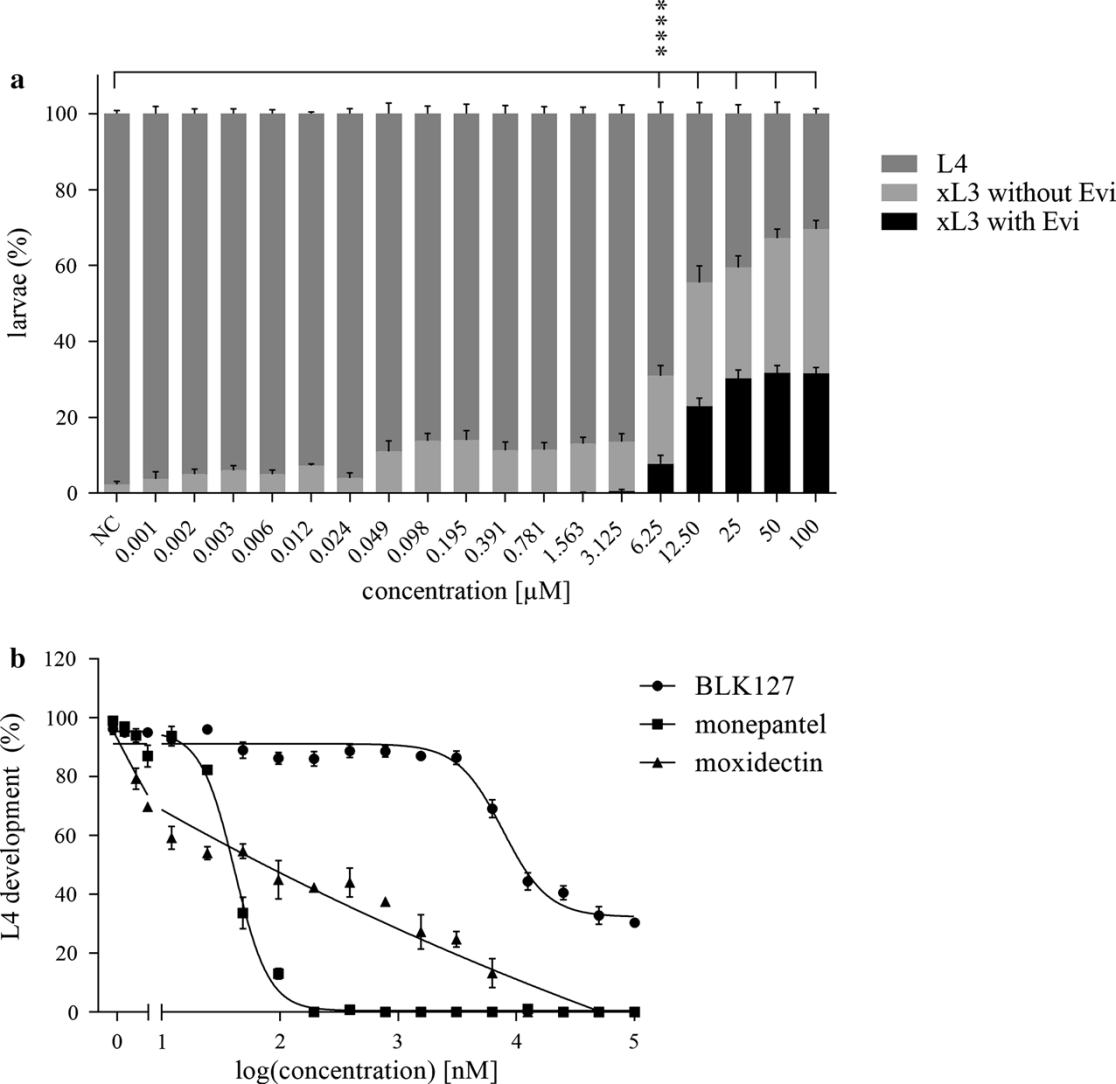
Effect of the compound BLK127 on the development of exsheathed third-stage larvae (xL3) to the fourth-stage (L4) after 7 days. **a** Percentage of L4, xL3 with/without evisceration (Evi) phenotype with reference to a negative (untreated) control (NC) LB* + 0.5% DMSO. **b** Dose–response curve for inhibition of L4 development. L4 development was assessed by light microscopy. **** denotes significance: *P* < 0.0001 compared to the NC

In the primary screen, compound BLK127 significantly (one-way ANOVA and Dunnett’s multiple comparison test: *F*_(4,13)_ = 257.5, *P* < 0.0001) reduced L4 development at a concentration of 20 µM, with 52% of treated worms developing to L4s within 7 days (Fig. 3). In a dose–response assay, the first concentration at which Evi phenotype was detected with the significant difference (one-way ANOVA and Dunnett’s multiple comparison test: *F*_(18,114)_ = 144.0, *P* < 0.0001) from the untreated control was 6.25 µM (Fig. 4a); at this concentration, 25% of xL3s had an Evi phenotype. At the highest tested concentration of 100 µM, 30% of larvae developed to the L4 stage, and 46% of the remaining xL3s had the Evi phenotype. From the highest concentration to 25 µM, the percentages of xL3s with/without the Evi phenotype were similar. From the dose–response curves, the IC_50_ values for the inhibition of L4 development by compound BLK127 and monepantel were 7.98 ± 0.68 µM and 0.04 ± 0.01 µM, respectively (Fig. 4b). The Evi phenotype was induced only during treatment of xL3s, but not L4s.

Compound HBK4 induced a particular ‘curved’ xL3 phenotype in the primary screen. Irrespective of the ‘curved’ phenotype, L4 development did not appear to be inhibited, as most xL3s (95%) developed to L4s when exposed to HBK4 at a concentration of 20 µM (Fig. 3). However, the length of L4s that developed from xL3s exposed to HBK4 for 7 days (693 ± 15 µm) was significantly shorter (unpaired t-test: *t*_(32)_ = 2.428, *P* = 0.0210) than the untreated control group (measuring 745 ± 14 µm); nonetheless, the width of HBK4-treated L4s (18.3 ± 0.3 µm) was not significantly different from the untreated control group (18.9 ± 0.6 µm) (unpaired t-test: *t*_(32)_ = 0.9374, *P* = 0.3556). The ‘curved’ phenotype was also observed in the dose–response motility assays (for both xL3s and L4s) at concentrations beyond 6.25 µM (i.e. 100 µM, 50 µM, 25 µM and 12.5 µM). The most remarkable alterations associated with larvae with the ‘curved’ phenotype were observed in the intestine and the cuticle of L4s after 72 h of exposure to compound HBK4. By comparison with untreated controls, affected larvae had a disorganised internal structure and the presence of variable numbers of vacuoles of distinct sizes within the intestinal cells in the middle part of the body. No obvious alterations in the cephalic region, including pharynx and oesophagus, were detected by light microscopy. Another marked alteration was cuticular wrinkling, which commenced in the transition between oesophagus and intestine and continued to the distal part of the gut. Similar morphological changes were also observed in L4s treated with monepantel at concentrations of between 25 µM and 100 µM. However, in the primary screen, monepantel resulted in a particular ‘coiled’ phenotype (Fig. 2; cf. [11, 13]). With respect to untreated controls, it was observed that HBK4-treated L4s with cuticular wrinkling were still capable of a limited range of movement only within the head or tail region, whereas larvae with vacuoles were immobile.

### Effects of two identified active compounds on inhibiting xL3 and L4 motility in a dose–response assay

**Fig. 5:**
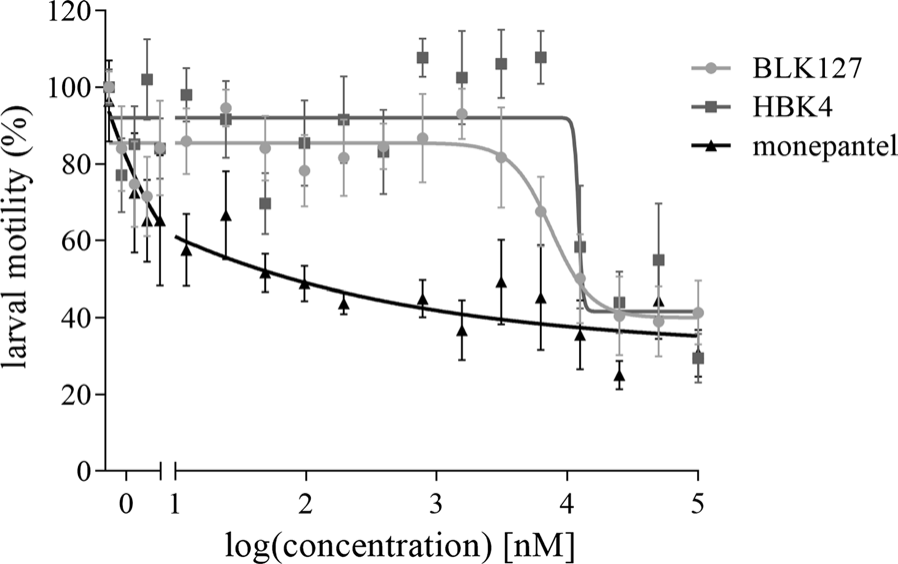
Dose–response curves for test compounds BLK127 and HBK4 on larval stage of *Haemonchus contortus in vitro*. Inhibition of larval motility after 7 days of exposure of exsheathed third-stage larvae to test or control (monepantel) compounds. Each data point represents the mean of three experiments (± standard error of the mean, SEM)

**Fig. 6:**
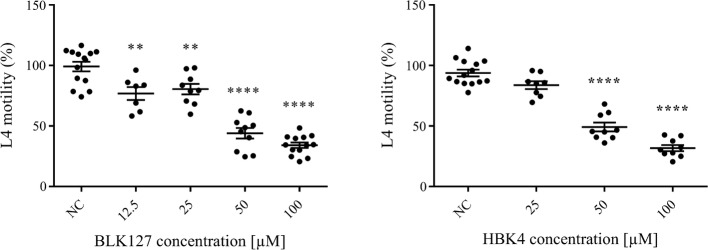
Assessment of the effect of test compounds BLK127 and HBK4 on inhibition of the fourth-stage larvae (L4) motility at selected concentrations at 72 h compared with negative control (LB* + 0.5% DMSO; NC). Statistical significance in comparison to NC: **P < 0.01; ****P < 0.0001

Although compounds BLK127 and HBK4 did not significantly reduce xL3 motility, even at the highest concentration of 100 µM for 72 h, they did inhibit larval motility at 7 days, with IC_50_ values of 7.45 ± 1.76 µM and 12.17 ± 2.28 µM, respectively (Fig. 5). Compounds BLK127 and HBK4 reproducibly inhibited L4 motility at concentrations from 12.5 µM to 100 µM and from 25 µM to 100 µM, respectively. Significance between values determined by one-way ANOVA and Dunnett’s multiple comparison tests were: *P* = 0.0018 at 12.5 µM, *P* = 0.0051 at 25 µM, *P* < 0.0001 at 50 µM and 100 µM; *df* = 4, *F*_(4, 48)_ = 55.19 for compound BLK127; and *P* < 0.0001 at 50 µM and 100 µM; *df* = 3, *F*_(3, 36)_ = 92.22 for compound HBK4 (Fig. 6).

## Discussion

Of the 236 chemicals screened, two compounds, designated BLK127 and HBK4 (cf. Fig. 1), induced phenotypic alterations in larval stages of *H. contortus*.

The first compound, BLK127, induced an Evi phenotype, which is likely linked to an adverse effect of this compound on the excretory/secretory system (cf. [20]). The excretory pore is assumed to have an osmoregulatory function as well as a role in exsheathment [21]. The excretory system has been suggested as the source of the exsheathment fluid, which contains enzymes capable of degrading components of the cuticle [22]. In general, after hatching from an egg, *H. contortus* undergoes four larval moults from L1 to the adult stage [23, 24]. These steps appear to be tightly controlled by particular pathways and genes [25], and dysregulation thereof results in moulting defects and/or lethality [24].

The results for the L4 development assay after 7 days revealed significantly less L4s following exposure to BLK127 in relation to the untreated controls. It appears that the process of moulting from xL3 to the L4 stage is (partially) suppressed, and it was observed that most larvae retained their previous cuticle. It was suggested that pathways that govern exsheathment and development are distinct, although the external stimuli for these processes appear to be shared, to some extent, particularly in early life-cycle stages [26]. The process of moulting and its modulatory factors have been well-studied in the free-living nematode *Caenorhabditis elegans*. The factors that control nematode moulting are still relatively elusive, but there is evidence linking steroid hormones and cholesterol to moulting defects in mutant *C. elegans* [27, 28]. As would be expected, a number of genes encoding both structural components of the cuticle and enzymes that modify cuticular proteins have been identified in screens for moulting-defective mutants [24].

Compound HBK4 was the second ‘hit’ compound from the random chemical collection, ‘Kurz-box’, and is a benzimidazole derivative. Benzimidazoles are a class of widely-used anthelmintic agents with a relatively broad spectrum of activity against gastrointestinal worms [1]. Even though the Haecon-5 strain of *H. contortus* is partially benzimidazole resistant (cf. [11, 17]), compound HBK4 induced a ‘curved’ phenotype at the L4 stage (Fig. 2). This phenotype has not been observed previously, although other phenotypes have. For instance, two pyrazole-5-carboxamide derivatives have been reported to induce a ‘straight’ phenotype after 72 h of exposure of xL3 [29], contrasting a ‘coiled’ phenotype induced by monepantel [11].

The present results showed that compound HBK4 was markedly more potent on L4s than xL3s. Similar findings have been made for other ‘hit’ compounds, including tolfenpyrad [11], SN00797439 [14] and deguelin [15]. This difference in potency might relate, for example, to a difference in the extent of compound uptake (*via* the mouth and alimentary tract) or metabolism between these two developmental stages. Nevertheless, further light microscopic examination of this non-wild type larval phenotype revealed changes in the cuticle and internal structures.

The cuticle is important in that it gives a worm its shape, provides protection and allows some metabolic exchanges with the surrounding environment [30]. Therefore, we assume that the structural cuticular disturbances observed here might lead to a possible impairment in the movement of the worm and, ultimately, death of the worm. Studies of *H. contortus* have also identified cuticular damage by scanning electron microscopy [14, 31]. Regarding the second observed morphological change, the formation of vacuoles had been recorded in early experiments [19]. The author of the latter study observed that L3s exposed to unfavourable conditions, such as desiccation, light or heat, developed vacuoles mainly in the intestinal cells at different time points, depending on the conditions and media used. The occurrence of many such vacuoles indicates that larvae were nearing death [19], which is consistent with the present study where larvae with vacuoles were immotile. The disorganised internal structures of treated larvae indicate that the two compounds identified herein induce phenotypes that are not compatible with the life of a worm.

Future work could focus on further evaluating the phenotypic and pathophysiological changes in the worms as well as the mechanisms underlying these changes. The morphology of worms could be assessed directly and, in more detail, using confocal microscopy or scanning electron microscopy. Nowadays, the coherent anti-Stokes Raman scattering spectroscopy [32–34] enables the distribution of lipids to be assessed in a rapid and label-free manner; this method could explore compositional differences between treated and untreated larvae, mainly in the intestinal tract, where significant alterations were seen here.

## Conclusions

The results of the present study provide a sound basis for future work, aimed at identifying one or more new anthelmintics and their targets. The phenotypic alterations induced by compounds BLK127 and HBK4 in *H. contortus* might stimulate further pharmacological research, as there has been interest in finding new agents that interfere with moulting in nematodes [35]. Moreover, assessing the activity of these compounds against other socioeconomically important parasites and their biotransformation would be interesting.

## Additional file

**Additional file 1.** Synthesis and features of chemicals HBK4 and BLK127 in the present study.

## Abbreviations

IC_50_: half maximum inhibitory concentration
L4: fourth-stage larvae
LB: Luria Bertani medium
xL3: exsheathed third-stage larvae
