# Supplementary material for: Phenotypic screening of the ‘Kurz-box’ of chemicals identifies two compounds (BLK127 and HBK4) with anthelmintic activity in vitro against parasitic larval stages of Haemonchus contortus
Source: Parasit Vectors. 2019 Apr 30;12:191. doi: 10.1186/s13071-019-3426-7 (PMC6492431; doi:10.1186/s13071-019-3426-7)
Supplement: Supplementary file 1 — Additional file 1. Synthesis and features of chemicals HBK4 and BLK127 in the present study. [file 13071_2019_3426_MOESM1_ESM.docx]

**Additional file 1.** Synthesis and features of chemicals HBK4 and BLK127 in the present study.

**General procedures.** All solvents and chemicals were used as purchased without further purification. The progress of all reactions was monitored on Merck precoated silica gel plates (with fluorescence indicator UV_254_). Flash-purification was performed with a CombiFlash RF200 with the solvent mixtures specified in the corresponding experiment. Spots were visualized by irradiation with ultraviolet light (254 nm). Melting points (m.p.) were taken in open capillaries on a Stuart SMP11 or a Büchi M565 melting point apparatus and are uncorrected. Proton (^1^H) and carbon (^13^C) NMR spectra were recorded on a Bruker Avance 300 MHz or 600 MHz using DMSO-d_6_ as solvent. Chemical shifts are given in parts per million (ppm), (δ relative to residual solvent peak for ^1^H and ^13^C). High-resolution mass spectrometry (HRMS) analysis was performed using a UHR-TOF maXis 4G instrument (Bruker Daltonics, Bremen, Germany). The Purity of compounds was determined by high performance liquid chromatography (HPLC). Purity of all compounds was 95% or higher. Instruments: Elite LaChrom system [Hitachi L-2130 (pump) and L-2400 (UV-detector)]; column: Phenomenex Luna C-18(2) 5 µm particle size (250 mm × 4.6 mm), supported by Phenomenex Security Guard Cartridge Kit C18 (4.0 mm × 3.0 mm) or Knauer system equipped with an Optimas autosampler, P6.1 pumps and K-2600 UV-detector and Oven CT 2.1 using a column Knauer Eurospher II 100-5 C18 150x4 mm.

**1. HBK4**

Preparation of *N*‑(4‑(5‑(phenylsulfonamido)‑1*H*‑benzo[*d*]imidazol‑2‑yl)phenyl)benzenesulfonamide (**HBK4**).

To a solution of 2-(4-aminophenyl)-1*H*-benzo[*d*]imidazol-5-amine (**1)** (112 mg, 0.5 mmol) in dry THF (15 ml) and dry pyridine (3.5 ml), benzenesulfonyl chloride (221 mg, 1.25 mmol) was added dropwise over 5 min. The reaction was then refluxed for 5 h, followed by the evaporation of THF under reduced pressure. Water (10 ml) was added to the mixture and the reaction mixture was extracted with EtOAc (3 x 20 ml). The organic phases were combined and dried over Na_2_SO_4_. After evaporation of the solvent the crude product was purified by flash chromatography (n-hexane/EtOAc 0-100%) to yield 85 mg of beige crystals (0.17 mmol, 34%). 2-(4-Aminophenyl)-1*H*-benzo[*d*]imidazol-5-amine (**1**) was synthesized according to Feitelson et al. [1].

**HBK4:** mp: 155 °C.

^1^H NMR(600 MHz, DMSO-d_6_), δ (ppm): 12.67-12.73 (m, 1H), 10.64 (s, 1H), 10.05-10.15 (m, 1H), 7.95 (d, J = 7.7 Hz, 2H), 7.82-7.83 (m, 2H), 7.72 (m, 2H), 7.59- 7.62 (m, 1H), 7.54‑7.57 (m, 3H), 7.49-7.51 (m, 2H), 7.34-7.44 (m, 1H), 7.22-7.23 (m, 3H), 6.92 (br s, 1H). ^13^C NMR (150 MHz, DMSO-d_6_), δ (ppm): 103.7, 111.3, 111.7, 116.4, 117.7, 118.8, 119.5, 125.4, 126.6, 127.3, 129.0, 129.3, 132.4, 132.6, 133.0, 139.1, 139.3, 139.4, 151.0. HRMS/ESI [M + H^+^] calcd. for C_25_H_20_N_4_O_4_S_2_: 505.0999, found: 505.0997. HPLC (isocratic elution with 70% MeOH in H_2_O, flowrate: 1.0 ml/min) retention time: 5.50 min, purity: 98.4% [2].

**2. BLK127**

Preparation of *N*-(benzyloxy)-4-(pentyloxy)benzamide (**BLK127**).

To a solution of 4-pentyloxybenzoic acid (**2**) (425 mg, 2 mmol) in dry DMF (10 ml) HATU (845 mg, 2.2 mmol) and DIPEA (313 mg, 2.4 mmol) were added. The solution was then stirred for 0.5 h at 60 °C followed by the addition of *O*‑benzylhydroxylamine (246 mg, 2 mmol) (**3**). The mixture was stirred for 5 h at 60 °C and for 60 h at RT. The solvent was evaporated under reduced pressure and then 50 ml ethyl acetate and 15 ml of a NaHCO_3_ solution was added. The mixture was extracted with ethyl acetate and washed 3 times with 15 ml of a NaHCO_3_ solution and 1 time with 15 ml brine. The organic layer was dried over Na_2_SO_4_. After evaporation of the solvent the crude product was purified by flash chromatography (n-hexane/EtOAc 0-100%) to yield 523 mg of white crystals (1.67 mmol, yield 83%).

**BLK127**: mp: 114 °C

^1^H NMR (300 MHz, DMSO-d_6_) δ 11.61 (s, 1H), 7.71 (d, J = 8.8 Hz, 2H), 7.51 – 7.32 (m, 5H), 6.99 (d, J = 8.9 Hz, 2H), 4.91 (s, 2H), 4.01 (t, J = 6.5 Hz, 2H), 1.72 (p, J = 6.7 Hz, 2H), 1.38 (tt, J = 9.0, 4.6 Hz, 4H), 0.90 (t, J = 7.1 Hz, 3H). ^13^C NMR (75 MHz, DMSO-d_6_) δ (ppm): 13.8, 21.8, 27.6, 28.2, 67.6, 76.9, 114.0, 124.1, 128.2, 128.2, 128.8, 128.8, 136.0, 161.2, 164.1. HRMS/ESI [M + H^+^] calcd. for C_19_H_24_NO_3_: 314,1751, found: 314,1750. HPLC (linear gradient; 10% to 100% acetonitrile +0.1% TFA in H_2_O +0.1% TFA in 20 min, flow rate of 1 mL/min) retention time: 15.43 min, purity: 99.5%

**References**

1. Feitelson BN, Mamalis P, Moualim RJ, Petrow V, Stephenson O, Sturgeon B. Some benzimidazole derivatives. J Chem Soc. 1952;448:2389–2398.
2. Konzuch S. Strukturoptimierung von antiplasmodial aktiven Pyrrolopyrazol-, Benzimidazol- und Fosmidomycin-Derivaten. Doctoral dissertation. Heinrich Heine Universität Düsseldorf, Germany. 2016.
